# ARL6IP5 in cancers: bidirectional function and therapeutic value

**DOI:** 10.1038/s41417-025-00903-x

**Published:** 2025-04-19

**Authors:** Zenan Hu, Hanxun Yue, Liang Qiao

**Affiliations:** 1 The First School of Clinical Medicine, Lanzhou University, Lanzhou, China; 2 Gansu Province Clinical Research Centre for Digestive Diseases, The First Hospital of Lanzhou University, Lanzhou, China; 3 Department of Gastroenterology, The First Hospital of Lanzhou University, Lanzhou, China; 4 Storr Liver Centre, Westmead Institute for Medical Research, The University of Sydney and Westmead Hospital, Westmead, NSW Australia

**Keywords:** Biomarkers, Targeted therapies

## Abstract

ARL6IP5 (ADP-ribosylation-like factor 6 interacting protein 5) plays an important role in a variety of physiological or pathological processes, including in cancers. However, the biological roles of ARL6IP5 in cancers are controversial. In this mini-review, we summarized the current understanding on the role of ARL6IP5 in cancers, particularly in the progression of chronic hepatitis virus-related hepatocellular carcinoma, as well as the potential values of ARL6IP5 in cancer therapy.

## Introduction

Cancer is a leading cause of death worldwide, creating a significant health, social, and economic burdens [1–3]. Cancer-related deaths have increased by 25.4% worldwide per year between 2007 and 2017 [4]. The absolute disability-adjusted life years of cancer have increased by 20.9% during 2010 to 2019 [5]. Understanding the molecular pathogenesis of cancers is a pre-requisite for developing more efficient anti-cancer therapies.

Accumulation of multiple genetic alterations and the complex interactions between oncogenes and tumor suppressor genes play important roles in cancer development [6–9]. Identification of key driver genes for cancer formation can facilitate the development of targeted therapies.

ADP-ribosylation-like factor 6 interacting protein 5 (ARL6IP5, also known as JWA, DER11, GTRAP3-18, or HSPC127) was initially cloned from all-trans-retinoic acid (ATRA)-treated human bronchial epithelial cells, but was later found to be ubiquitously expressed in most of the human tissues [10]. The gene encoding ARL6IP5 is located at chromosome 3p, and its protein is localized to endoplasmic reticulum (ER) and Golgi apparatus. ARL6IP5 is a homolog of *Drosophila* PRAF2, a small protein from the prenylated Rab acceptor family that plays a role in ER-to-Golgi transport [11]. The rodent homologs of ARL6IP5, addicsin in mice and glutamate transporter-associated protein 3-18 (GTRAP3-18) in rats, are abundantly present in the brain and play important roles in neuronal differentiation and glutathione regulation [12–16].

ARL6IP5 is involved in the regulation of multiple physiological and pathological processes, such as glutamate transportation, oxidative stress, autophagy, and DNA damage repair [17–22]. Publicly available Gene Expression Profiling Interactive Analysis (GEPIA) dataset (gepia.cancer-pku.cn) reveals an overexpression of ARL6IP5 in the cancer tissues originating from lymph, brain, kidney, blood, pancreas, skin, and thymus, relative to their respective normal tissues. However, in other cancer tissues including those from bladder cancer, squamous carcinoma of the cervix, squamous cell lung carcinoma, endometrial cancer, and sarcoma of the uterus, a down-regulation of ARL6IP5 was observed. These data indicate that the roles of ARL6IP5 in cancers may be bidirectional and context-dependent.

As shown in Table 1, the role of ARL6IP5 in human cancers is mixed and contradictory: it functions as a tumor suppressor in most cancers, but in some cancers, it acts as an oncogene. The biological functions of ARL6IP5 may depend on many factors such as tumor microenvironment and etiological factors. For example, in liver cancer, ARL6IP5 is more involved in the pathogenesis of hepatitis c virus (HCV)-related cancers.

**Table 1:** Representative studies on the roles of ARL6IP5 in cancers.

Author	Year	Origin of cancer	Relative Molecules	Functional Role	References
Huang S	2006	Blood	–	Tumor-suppressing	[42]
Chen X	2015	Breast	–	Tumor-suppressing	[56]
Xu L	2018	Breast	CXCR4	Tumor-suppressing	[30]
Zhai Z	2022	Breast	JAC1 YY1	Tumor-suppressing	[55]
Mao W	2006	Cervix	ATRA	Tumor-suppressing	[31]
Lin J	2014	Esophagus	–	Tumor-Suppressing	[34]
Chen H	2007	Liver	F-actin	Tumor-suppressing	[29]
Wu X	2014	Liver	FAK RhoA MMP-2	Tumor-suppressing	[23]
Li Y	2015	Lung	EGCG topoisomerase IIα	Tumor-suppressing	[24]
Kim J	2022	Ovary	–	Tumor-suppressing	[64]
Wu Y	2014	Pancreas	–	Tumor-suppressing	[54]
Lu J	2013	Skin	ING4	Tumor-Suppressing	[33]
Liu X	2012	Stomach	p53	Tumor-suppressing	[25]
Wang S	2012	Stomach	XRCC1	Tumor-suppressing	[76]
Ye Y	2013	Stomach	MDM2	Tumor-suppressing	[26]
Lu J	2013	Stomach	ILK	Tumor-suppressing	[32]
Chen Y	2014	Stomach	MMP-2	Tumor-Suppressing	[27]
Xu W	2014	Stomach	CK2	Tumor-suppressing	[65]
Qiu D	2018	Stomach	RNF185	Tumor-suppressing	[66]
Wang W	2020	Stomach	XCCR1	Tumor-suppressing	[28]
Li C	2007	Bladder	–	Oncogenic	[37]
Shen Q	2005	Blood	–	Oncogenic	[41]
Zhu T	2006	Blood	p53	Oncogenic	[48]
Li Z	2013	Blood	–	Oncogenic	[39]
Chen R	2005	Breast	–	Oncogenic	[49]
Wang W	2013	Lymph	–	Oncogenic	[28]
Romanuik T	2009	Prostate	–	Oncogenic	[35]
Cunha I	2010	Prostate	–	Oncogenic	[36]
Gong Z	2012	Skin	Elk1	Oncogenic	[53]
Shen Q	2007	Blood	–	Bidirectional	[38]

In this article, we aim to provide an overview of the consensus and controversies of the roles of ARL6IP5 in human cancers. The review provides valuable insights in the search for novel therapeutic strategies for cancers.

## ARL6IP5 plays bidirectional roles in different cancers

ARL6IP5 is expressed in many human tissues where it functions as a tumor suppressor gene. For instance, functional studies have shown that down-regulation of ARL6IP5 in hepatocellular carcinoma (HCC) and non-small cell lung cancer can promote tumor invasion and predict a poor prognosis [23, 24]. In gastric cancer, ARL6IP5 deficiency together with p53 mutation promotes tumor invasion and metastasis [25]. Combination of murine double minute 2 (MDM2, a negative regulator for p53) overexpression and ARL6IP5 down-regulation led to a shorter overall survival in patients with gastric cancer [26]. Mechanistic studies have shown that ARL6IP5 insufficiency and up-regulation of matrix metalloproteinase-2 (MMP-2) can increase tumor micro vessel density in gastric cancer [27]. Thus, ARL6IP5 has been regarded as an effective biomarker for gastric cancer [28]. The tumor suppressor roles of ARL6IP5 have also been observed in other common cancers, including esophagus, liver cancer, breast cancer, cervical cancer, and skin cancer [29–34].

However, ARL6IP5 also functions as an oncogene in some cancers [35, 36]. Three novel functional genetic polymorphisms of ARL6IP5, namely -76GC, 454CA, and 723TG, have been identified to contribute to the development of bladder cancer [37]. The functional variations of the -76C allele are correlated to the significantly increased odds of leukemia, whereas those of the 723 G allele are associated with markedly decreased odds of leukemia [38]. A meta-analysis showed that increased expression of ARL6IP5 is related to worse overall survival and event-free survival of leukemia patients, and ARL6IP5 overexpression is an independent risk factor of poor survival in leukemia patients [39]. ARL6IP5 overexpression is also strongly associated to Burkitt lymphoma progression [40]. The mechanisms of how ARL6IP5 exerts a tumor suppressor or oncogenic effects in cancers will be further discussed below.

## Functional mechanisms and regulatory network of ARL6IP5

The differential biological functions of ARL6IP5 across different cancers may be attributed to multifactorial mechanisms [41, 42]. Under the physiological conditions, GTRAP3-18 (one of the homologous proteins of ARL6IP5) was found to suppress excitatory amino-acid carrier 1 (EAAC1)-mediated glutamate transport by impairing its affinity to the substrate, reducing the L-glutathione level at the plasma membrane, or delaying the exit of EAAC1 from the endoplasmic reticulum [13, 17, 18, 43, 44]. GTRAP3-18 was also shown to negatively regulate excitatory amino acid transporter 3 **(**EAAT3) functions. ARL6IP5 can promote apoptosis of mouse embryonic cells, which is directly targeted by CCAAT/enhancer binding protein (C/EBP) alpha. C/EBP alpha can bind and activate the ARL6IP5 promoter [45]. In the early secretory pathway, ARL6IP5 inhibits Rab1, thus reducing the transportation efficiency of ER-to-Golgi [46]. Under the pathological conditions, ARL6IP5 plays important role in oxidative stress. It was found that ARL6IP5 is an important signaling molecule in hydrogen-peroxide-induced cell injury [47]. It enhances intracellular defense mechanisms against oxidative stress in myelogenous leukemia cells, participates in the signaling pathways of DNA damage and repair, especially excision repair [48, 49]. In breast cancer, ARL6IP5 is involved in the estrogen receptor-related signal transduction pathways [21].

Mitogen-activated protein kinases (MAPK) signaling pathway is one of the most ancient signaling pathways that participate in many physiological processes. It converts extracellular stimuli into cellular responses, which can be divided into seven groups, and the most extensively studied mammalian MAPK groups are ERK1/2, JNK, and p38 isoforms [50, 51]. In some cancers, ARL6IP5 exerts its roles through regulating the activity of MAPKs. For example, Chen H et al. showed that ARL6IP5 inhibits tumor cellular migration via activating MAPK cascades and rearranging the F-actin cytoskeleton [29]. ARL6IP5 up-regulates the activity of E2F transcription factor 1 (E2F1) via activating MAPK signaling pathway and subsequently the activation of X-ray repair cross complementing 1 (XRCC1). Additionally, ARL6IP5 protects the XRCC1 protein from ubiquitination and degradation by proteasomes [52]. In several cancers, such as skin cancer, pancreatic cancer, and breast cancer, the tumor suppressive role of ARL6IP5 was found to be mediated via its inhibitory effects on MAPK signaling pathway and JNK pathways [53–56].

**Fig. 1:**
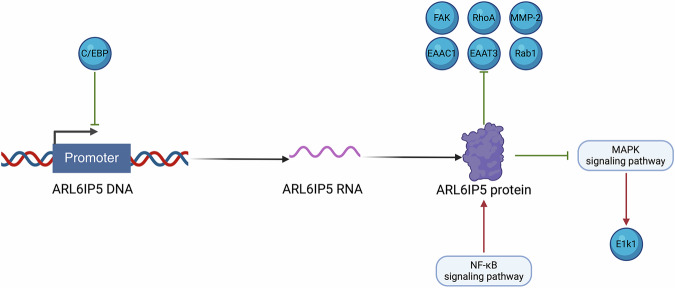
Regulatory network of ARL6IP5 in human cancer.

Human NF-κB repressing factor (NKRF) is a negative regulator for NF-κB. Using a genome-wide expression profile analysis, Sun Y et al. validated that knockdown of NKRF in HEK293 cells led to a significant up-regulation of ARL6IP5, suggesting ARL6IP5 may play an important role in the NF-κB signaling cascade [57] (Fig. 1).

It is well-known that PI3K-Akt-mTOR signaling pathway is closely related to cancers. In FAK-PI3K-Akt-mTOR cascade, the deficiency of ARL6IP5 can increase the number of neurons and enhance the long-term potentiation induction in the hippocampal dentate gyrus, thereby leading to spatial cognitive potentiation [58]. Through this signaling pathway, ARL6IP5 deletion in astrocytes exacerbates dopaminergic neurodegeneration by decreasing glutamate transporters in mice [59]. From these findings, we speculate that ARL6IP5 may regulate cancers via PI3K-Akt-mTOR signaling pathway.

## Therapeutic potentials of ARL6IP5 in cancers

The potential application of ARL6IP5 as a therapeutic target in cancer therapy has been reported. Studies on N-methyl-N’-nitro-N-nitrosoguanidine (MNNG) have inspired researchers to harness the tumor-suppressing effects of ARL6IP5 to conquer cancers. MNNG treatment can activate nuclear transcription factor binding to the ARL6IP5 proximal promoter, thereby triggering apoptosis [60]. Arsenic trioxide is a standard therapy for refractory acute promyelocytic leukemia, and it can induce apoptosis in a variety of malignant cells. Arsenic trioxide up-regulates the expression of ARL6IP5 by stimulating the production of reactive oxygen species in a dose-dependent manner, and ARL6IP5 induces apoptosis and loss of mitochondrial transmembrane potential in breast cancer cells [61]. Arsenic trioxide-induced apoptosis depends in part on tubulin polymerization. The activation of p38 MAPK contributes to ARL6IP5-promoted tubulin polymerization which also improves the sensitivity of breast cancer cells to arsenic trioxide [62]. Cadmium chloride treatment can also promote apoptosis, which is attributed to the up-regulation of ARL6IP5 and its promoter activity [63]. In ovarian cancer, ARL6IP5 appeared to exert a tumor suppressive role, and as such, recombinant ARL6IP5 protein was demonstrated to sensitize the ovarian cancer cells to cisplatin [64]. Similarly, ARL6IP5 was shown to reverse cisplatin-resistance in gastric cancer [65]. Other studies have shown that targeting the upstream molecules of ARL6IP5 maybe an effective cancer therapeutic strategy. In this regard, inhibition of ring finger protein 185 **(**RNF185) was found to inhibit the metastasis of gastric cancer [66].

However, because ARL6IP5 allows cells to escape from DNA damage, strategies enhancing sensitivity of tumors to antitumor drugs by downregulating ARL6IP5 have been reported [52]. In this regard, inhibition of ARL6IP5 may enhance the sensitivity of certain anticancer agents. For example, inhibiting ARL6IP5-XRCC1-mediated DNA single-strand-break repair (SSBR) could reverse the resistance of ovarian cancer cells to Cx-platin-Cl and Cx-DN604-Cl (two Pt(IV) prodrugs), and restore the sensitivity of ovarian cancer cells to cisplatin [67]. Cis-wog, a cytotoxic agent, has been shown to improve the antitumor activity of its corresponding Pt(II)-based drugs and reverse resistance to by inhibiting ARL6IP5-mediated SSBR in lung adenocarcinoma cells [68].

Considering the controversial expression patterns and biological functions of ARL6IP5 across different cancers, the therapeutic potential of this gene needs more extensive studies. ARL6IP5 has tumor-suppressing effects, such as promoting apoptosis. ARL6IP5 can also make tumor cells resistant to drugs via promoting SSBR. Therefore, both up-regulation and down-regulation of ARL6IP5 may be utilized as strategies in cancer therapy (Table 2). At present, researchers have taken the first step toward specifying the cancer therapeutic strategies surrounding ARL6IP5. Extensive research is needed to clarify the context-dependent role of ARL6IP5 in different cancers.

**Table 2:** Strategies using ARL6IP5 as a therapeutic target.

Materials	Regulation	Mechanism	Ref.
arsenic trioxide	upregulation	inducing apoptosis and loss of mitochondrial transmembrane potential, promoting tubulin polymerization	[61, 62]
recombinant ARL6IP5 protein	upregulation	suppressing DNA damage repair	[64]
Cx-platin-Cl/Cx-DN604-Cl	downregulation	suppressing DNA damage repair	[67]
cis-wog	downregulation	suppressing DNA damage repair	[68]

## ARL6IP5 plays an important role in HCV-related HCC

Expression profiling shows that most of cancers, including HCC, express high level of ARL6IP5. However, the functions of ARL6IP5 are likely to be cell- and context-dependent. Moreover, microbiota can modulate cancer development by shaping the immune system [58]. For instance, persistent Helicobacter pylori infection is significantly associated with gastric cancer and lymphoma. Hepatitis B (HBV) or C (HCV) viruses are known risk factors for HCC [69]. HCV can synergistically promote HCC development with other risk factors such as alcohol, HBV X protein, and aflatoxin B1 [70]. We previously showed that ARL6IP5 is involved in the pathogenesis of HCV-related liver cancer, and this was supported by other studies [17, 71, 72]. In HCV-infected liver, ARL6IP5 increases the levels of oxidative stress markers such as 8-oxo-dG, 4-hydroxynonenal, and malondialdehyde [10, 72–74].

It is also noteworthy that ARL6IP5 also acts as a tumor suppressor in HCC. ARL6IP5 negatively regulates MMP-2 and FAK, which are factors facilitating cell attachment, motility, and invasion [23]. The tumor suppressive role of ARL6IP5 in liver cancer has also been reported, where ARL6IP5 was shown to inhibit HCC growth by inhibiting MAPK signaling pathway [75].

**Fig. 2:**
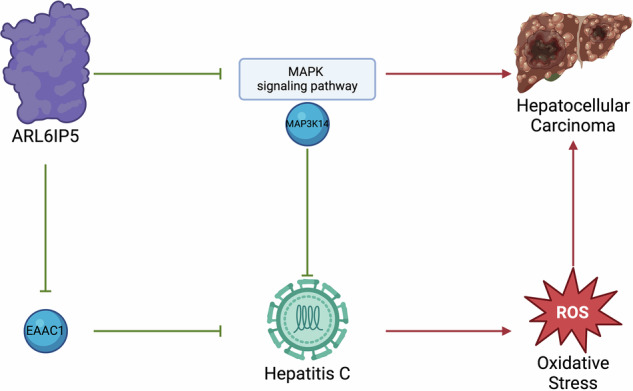
Possible role and underlying mechanisms of ARL6IP5 in HCC. The footnote of Fig. 2: ARL6IP5 inhibits HCC by suppressing MMP-2, FAK, and MAPK signaling pathway. However, ARL6IP5 can promote HCV replication by inhibiting EAAC1, and thus promote HCC. ARL6IP5 also enhances oxidative stress in HCV-infected liver, thereby increasing the risk of HCC. Meanwhile, MAPKs analogs suppress HCC via inhibiting HCV replication.

These studies indicate that there is a complex regulatory network among HCV, ARL6IP5 and HCC (Fig. 2). First, ARL6IP5 inhibits the development of HCC by inhibiting MMP-2, FAK and MAPK signaling pathway. However, ARL6IP5 can promote HCV replication by inhibiting EAAC1, and thus promote HCC. ARL6IP5 also enhances oxidative stress in HCV-infected liver, thereby increasing the risk of HCC.

It is likely that ARL6IP5 may play different or even conflicting roles in HCC under different microenvironments. More studies are needed to elucidate the role of ARL6IP5 and its therapeutic potential in HCC.

## Summary and conclusions

ARL6IP5 is abnormally expressed at different levels across different human cancers, hence, its biological role in different cancers may vary. The complex nature of ARL6IP5 is also reflected in the fact that it may exert both tumor-suppressing and oncogenic roles in the same cancer type. The biological functions of ARL6IP5 cannot be deduced based on its expression level. For instance, in gastric cancer, ARL6IP5 is significantly downregulated in cancerous tissues compared to matched non-cancerous mucosa. Despite this down-regulation, conditional ARL6IP5-knockout mice do not show spontaneous tumor formation [76]. This suggests that the biological function of ARL6IP5 in a given cancer type may be highly dependent on the tumor microenvironment, emphasizing its complex, context-dependent roles in cancer progression. As such, developing ARL6IP5 into a therapeutic target is likely premature. Further research is essential to unravel the precise mechanisms by which ARL6IP5 interacts with other molecules, signaling pathways, and tumor microenvironment. Understanding how ARL6IP5 influences tumorigenesis in different contexts will be critical for developing new, safe, and effective therapeutic strategies.

## Future perspectives

Studies on the roles of ARL6IP5 in cancers are still scarce, and the existing data do not entirely reveal the functional mechanisms and regulatory network of ARL6IP5. The dual role of ARL6IP5 in cancers implies that the biological roles of ARL6IP5 in different cancers may be context-dependent, and tumor microenvironments may be an important contributor therein. In liver cancer in particular, considering the potential importance of ARL6IP5 in the hepatitis-related HCC, and HBV and HCV are still major causes for HCC (currently worldwide, approximately 60% of new HCC cases can be attributed to chronic HBV infection) [77–80], further studies on the precise roles of ARL6IP5 in the pathogenesis of liver cancer are warranted.
